# Efficacy of teriparatide and factors for the collapse of femoral head following femoral curved varus osteotomy

**DOI:** 10.1093/jhps/hnaf005

**Published:** 2025-02-06

**Authors:** Shunichi Yokota, Tomohiro Shimizu, Hotaka Ishizu, Yutaro Sugawara, Yusuke Ohashi, Tsuyoshi Asano, Daisuke Takahashi, Norimasa Iwasaki

**Affiliations:** Department of Orthopedic Surgery, Faculty of Medicine and Graduate School of Medicine, Hokkaido University, Kita-15, Nish-7, Kita-ku, Sapporo 060-8638, Japan; Department of Orthopedic Surgery, Faculty of Medicine and Graduate School of Medicine, Hokkaido University, Kita-15, Nish-7, Kita-ku, Sapporo 060-8638, Japan; Department of Orthopedic Surgery, Faculty of Medicine and Graduate School of Medicine, Hokkaido University, Kita-15, Nish-7, Kita-ku, Sapporo 060-8638, Japan; Department of Orthopedic Surgery, Faculty of Medicine and Graduate School of Medicine, Hokkaido University, Kita-15, Nish-7, Kita-ku, Sapporo 060-8638, Japan; Department of Orthopedic Surgery, Faculty of Medicine and Graduate School of Medicine, Hokkaido University, Kita-15, Nish-7, Kita-ku, Sapporo 060-8638, Japan; Department of Orthopedic Surgery, Faculty of Medicine and Graduate School of Medicine, Hokkaido University, Kita-15, Nish-7, Kita-ku, Sapporo 060-8638, Japan; Department of Orthopedic Surgery, Faculty of Medicine and Graduate School of Medicine, Hokkaido University, Kita-15, Nish-7, Kita-ku, Sapporo 060-8638, Japan; Department of Orthopedic Surgery, Faculty of Medicine and Graduate School of Medicine, Hokkaido University, Kita-15, Nish-7, Kita-ku, Sapporo 060-8638, Japan

## Abstract

Osteonecrosis of the femoral head (ONFH) often leads to femoral head collapse, which makes joint preservation challenging. Although curved varus osteotomy (CVO) is an effective surgical method for joint preservation in young ONFH patients, there are some cases where it cannot prevent femoral head collapse. This research aims to evaluate the usefulness of teriparatide (TPD) in bone healing and preventing femoral head collapse in CVO-treated ONFH patients. This retrospective study included 48 patients (56 hips) diagnosed with ONFH, categorized into three groups: glucocorticoid-associated ONFH with TPD treatment (GCs + TPD), glucocorticoid-associated ONFH (GCs), and alcohol- associated ONFH (Alc). No significant differences were found in terms of surgical details, stage, conversion to total hip arthroplasty (THA), and clinical scores. The GCs + TPD group showed a shorter bone union duration, reduced femoral head collapse, and a lower incidence of advanced collapse than the other groups. Lower BMI and TPD use were associated with a shorter duration of bone union. TPD and union duration were identified as factors contributing to the advanced collapse. In conclusion, TPD administration accelerates bone union at the osteotomy site and mitigates femoral head collapse after joint-preserving osteotomy. In addition, combining TPD with CVO may be a promising strategy for younger patients.

## Introduction

Osteonecrosis of the femoral head (ONFH) is a condition characterized by necrosis in a section of the femoral head resulting from diminished blood supply due to various factors [1]. ONFH occurs mostly in younger patients, and the collapse of the femoral head hinders activities of daily living (ADL), diminishes the quality of life (QOL), and often necessitates surgical intervention [2, 3]. Although total hip arthroplasty (THA) is a commonly employed surgical strategy for advanced ONFH cases featuring femoral head collapse [4], preserving the joint is generally favored in younger patients due to concerns over potential future revisions [5]. Femoral curved varus osteotomy (CVO) is a useful and successful surgical procedure for joint preservation in young patients with ONFH in which the intact articular surface can be covered by more than one-third of the preoperative anteroposterior hip radiographs obtained at maximal abduction [6–8].

Despite undergoing CVO, some patients experience unsuppressed femoral head collapse, which makes joint preservation challenging [9–12]. The collapse of the femoral head is not simply caused by the structural fragility of the necrotic region but involves osteoclastic bone resorption that exceeds bone formation in the repair response at the boundary region between necrotic and healthy areas [13]. Although bisphosphonates, which mitigate osteoclast-mediated bone resorption, may lower the incidence of femoral head collapse in ONFH [14–16], their efficacy is disputed because conflicting studies indicate minimal influence [17, 18]. Teriparatide (TPD), a recombinant form of parathyroid hormone that enhances bone formation by osteoblasts [19], is superior to bisphosphonates in increasing bone mineral density and preventing fractures [20]. Beyond treating osteoporosis, TPD has demonstrated efficacy in skeletal repair for conditions such as osteonecrosis of the jaw [21–23] and fractures [24], and our previous research indicated its effectiveness in preventing femoral head collapse in ONFH [25]. However, there is little information regarding the efficacy of TPD for femoral osteotomy.

We hypothesized that TPD could both prevent the collapse of the femoral head and promote bone healing at the osteotomy site during CVO, contributing to joint preservation in ONFH cases. To address this hypothesis, this study aimed to compare bone healing at the osteotomy site and the progress of femoral head collapse among CVO-treated ONFH patients with and without TPD therapy and to investigate the progression factors associated with bone union at the osteotomy site and the collapse of the femoral head.

## Materials and methods

### Patients

This retrospective study was conducted in accordance with the ethical standards of the Declaration of Helsinki and was approved by our institutional review board (#019-0031). All patients were informed about the study and provided consent for its publication when they decided to undergo CVO surgery. A total of 48 patients (56 hips) diagnosed with ONFH who underwent CVO at our hospital between April 2012 and March 2022 with a minimum follow-up period of 24 months were eligible for inclusion in this study. The diagnosis of ONFH was confirmed by three orthopedic surgeons based on radiographic and MRI findings, following the diagnostic criteria reported by Sugano et al [26]. A comparative study was conducted by dividing the patients into three groups: glucocorticoid (GC)-associated ONFH treated with TPD for glucocorticoid-induced osteoporosis (GC + TPD), GC-associated ONFH without TPD administration (GC), and alcohol-associated ONFH (Alc). One patient with a history of GC administration and alcohol abuse was excluded. No patients in the Alc group were treated with TPD for osteoporosis in this study. In the GC + TPD group, the decision to initiate TPD as a treatment for GC-induced osteoporosis was made through consultations between the patient and their primary outpatient physician. The administration of TPD, 20 mg every day for 24 month, was then continued at the time of surgery based on approved indications.

### Surgical indication and technique of femoral curved varus osteotomy

**Figure 1.**
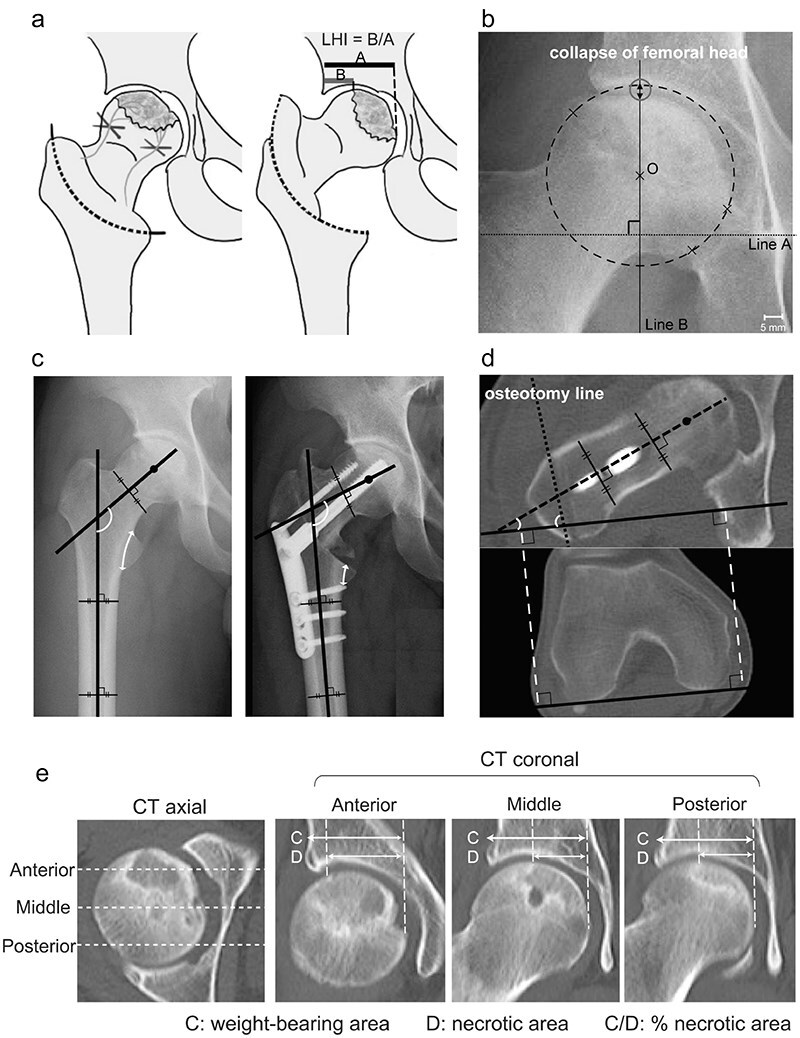
A detailed representation of the femoral curved varus osteotomy and associated measurements. (a) Demonstrates proximal femoral head fragment movement and how the lateral head index (LHI) is calculated. (b) Illustrates the radiographic indices used for measuring femoral head collapse. (c) Shows how the neck-shaft angle and varus angulation are determined, and how the percentage of the osteotomy line in the lesser trochanter is calculated. (d) Depicts the measurement of the anteversion of the osteotomy line and femoral neck based on the axial image of a CT scan. (d) Illustrates the necrotic area within weight-bearing area (% necrotic area) on anterior, middle, and posterior portions are measured.

The surgical indication for this procedure was the pain experienced by patients with ONFH who desired joint preservation surgery. Moreover, more than one-third of the weight-bearing area was required to have an intact articular surface (33% < intact ratio) based on preoperative anteroposterior radiographs of the hip taken during maximum abduction [6] (Fig. 1a). CVO was performed according to a technique described in a previous study [6, 8, 9]. First, the greater and lesser trochanters were exposed posteriorly with internal rotation of the hip joint. Next, an osteotomy guide was attached to a line from the top of the greater trochanter to a point 2–3 mm above the middle of the lesser trochanter under fluoroscopic guidance. After an intertrochanteric curved osteotomy was performed from the lesser to the greater trochanter according to the guide, the proximal femoral head fragment was moved into varus. The osteotomy area was stabilized using a plate and screws. Full weight-bearing was permitted 8 wk after surgery.

### Demographic data

Data on patients’ demography, including age, sex, body mass index (BMI), follow-up period, history of GC intake, alcohol abuse, use of TPD in the perioperative period as a treatment for steroid-induced osteoporosis, operative time, intraoperative bleeding, THA conversion after CVO, and Harris hip score (HHS), were collected from the medical records. Alcohol abuse was defined as the consumption of >400 mL of alcohol per week, which is a significant risk factor for ONFH [27]. Radiological stages and locations of osteonecrosis were assessed based on the Japanese Investigation Committee (JIC) staging and classification systems, respectively [28]. JIC stage 3A and 3B are further subdivisions of ARCO stage 3. Similarly, JIC types C1 and C2 are further subdivisions of the lateral type in the ARCO classification. These staging and classification systems demonstrate a consistent relationship.

### Measurement

Radiographs, MRI, and CT images of all patients were assessed using a picture archiving and communication system (PACS). The collapse of the femoral head, varus angulation obtained by osteotomy, the osteotomy line in the lesser trochanter, and bone union at the osteotomy site were evaluated on anteroposterior hip radiographs in neutral rotation using a similar technique throughout the study period. In the analysis of the collapse of the femoral head, concentric circles passing through three points in the healthy area set arbitrarily in the femoral head were drawn (Fig. 1b). The distance between the intersection of the circle and the outline of the femoral head onto the line that passes through the center of the femoral head (Line A and O in Fig. 1b), perpendicular to the line connecting the bilateral teardrops (Line B in Fig. 1b), was measured as the collapse of the femoral head. The varus angulation obtained by osteotomy was calculated as the difference in the femoral neck-shaft angle before and after surgery (Fig. 1c). The osteotomy line in the lesser trochanter was measured as the percentage of the distance from the distal end of the lesser trochanter to the osteotomy line relative to the distance from the distal to the proximal end of the lesser trochanter (Fig. 1c). Bone union was defined as the disappearance of the osteotomy line and the presence of continuity in the trabecular structure. To assess bone union, outpatient X-ray follow-ups were conducted at intervals of no longer than 1 month until bone union was determined to have been achieved. Anteversion of the osteotomy line and femoral neck was measured based on the axis of the femoral posterior condyle at the knee level on the axial CT image (Fig. 1d). The femoral head was divided into three portions—anterior, middle, and posterior—on the axial CT view (Fig. 1e). The necrotic area within the weight-bearing area was measured as a percentage of the necrotic area on the coronal view at the midpoint of each of these three portions (Fig. 1e).

### Blood test

Fasting blood samples were obtained to examine the biochemical markers of osteoporosis-related bone turnover, including the levels of intact type 1 procollagen-N-propeptide (P1NP) and tartrate-resistant acid phosphatase 5b (TRACP 5b). Because type I collagen-derived peptides, such as CTX-1 (cross-linked C terminal telopeptides of type I collagen) and NTX-1 (cross-linked N-telopeptide of type I collagen), are excreted through the kidneys, they can be affected by renal dysfunction; therefore, this study evaluated the serum levels of P1NP and TRACP-5b. Areal bone mineral density (BMD) in the lumbar spine (LS, L2–L4) and femoral neck was assessed by dual-energy X-ray absorptiometry (DXA; Discovery A, Hologic Japan, Inc, Tokyo, Japan). Bone turnover markers and BMD were investigated 1 wk before surgery.

### Statistical analysis

An estimation of sample size was performed to detect a clinically important collapse of the femoral head based on previous studies [29, 30]. This analysis determined that a total sample size of 36 patients (12 per group) would give 80% power to detect a significant difference (α = 0.05) in the collapse of the femoral head. Differences in continuous and non-continuous variables between the groups were analyzed using a one-way analysis of variance (ANOVA), followed by Tukey’s multiple-comparison procedure and Fisher’s exact test. Survival rates, with the endpoint defined as the collapse of the femoral head by >1 mm, were examined using the Kaplan–Meier method. Intergroup comparisons of the Kaplan–Meier data were performed using the log-rank test. Cox regression analysis was conducted to identify risk factors for the progression of femoral head collapse by >1 mm. Linear regression analysis was performed to determine independent factors associated with bone union at the osteotomy site. All statistical analyses were performed using GraphPad Software version 9.5.1 (528) (GraphPad Software Inc., San Diego, CA, USA). Differences below the probability level (*p* value) of 0.05 were considered statistically significant. Cohen’s kappa coefficient was calculated to evaluate the reproducibility and accuracy of the measurement of the progression of femoral head collapse by >1 mm.

## Results

### Patient Demographics

**Figure 2.**
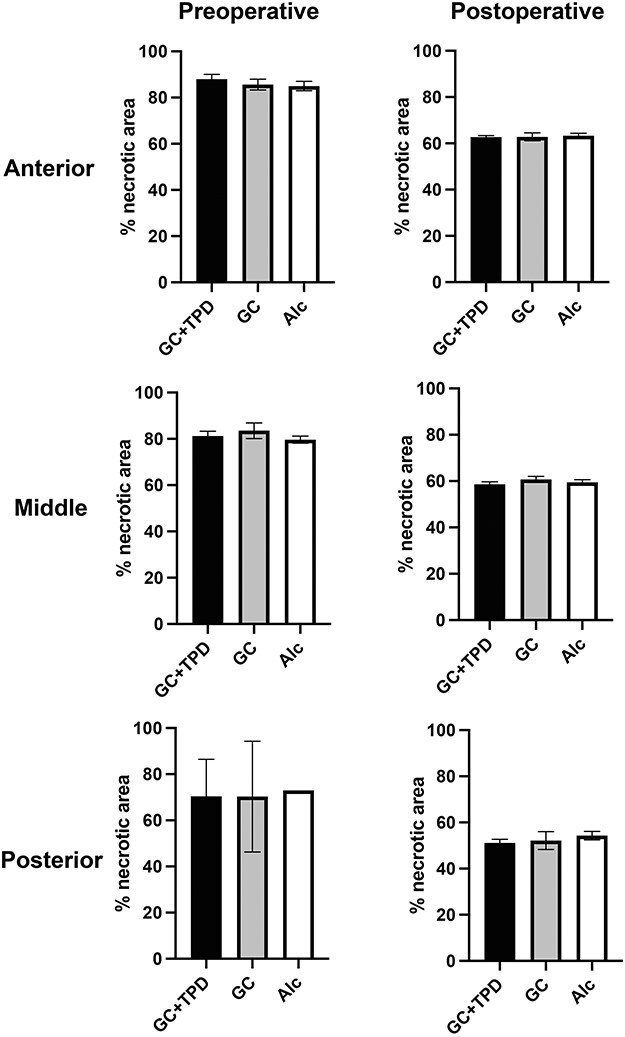
Comparison of % necrotic area in each group. There were no significant differences in the percentage of the necrotic area within the weight-bearing area (% necrotic area) among the three groups at any portions, at preoperative and postoperative periods.

Demographic and clinical data of patients revealed that there were no significant differences between the groups, except that the alcohol group was older than the GC group (Table 1). Although not statistically significant, the postoperative HHS was highest in the GC + TPD group. Table 2 presents the parameters related to surgical manipulation, including the operative time, intraoperative bleeding, and correction angle during osteotomy. No significant differences in these operative parameters were observed among the three groups. Postoperatively, all hips exhibited more than one-third of the weight-bearing area covered by an intact articular surface as determined by anteroposterior radiographs, and bone union was achieved at the osteotomy sites in all cases. There were no significant differences in the percentage of necrotic area among the three groups at any portion, either in the preoperative or the postoperative periods (Fig. 2)

**Table 1.** Demographics of the clinical population in each group.

	GC + TPD (N = 21)	GC (N = 17)	Alc (N = 18)	GC + TPD vs GSc	GC + TPD vs Alc	GC vs Alc
Age (years)	33.6 ± 2.5	28.0 ± 2.1	39.7 ± 1.4	*p* = 0.149	*p* = 0.107	***p* = 0.001**
Sex (male: female)	10:7	9:8	12:3	*p* = 0.730	*p* = 0.197	*p* = 0.108
BMI (kg/m^2^)	23.2 ± 0.80	21.6 ± 0.61	24.6 ± 0.97	*p* = 0.329	*p* = 0.479	*p* = 0.052
Follow up (months)	70.7 ± 4.69	51.5 ± 6.29	67.4 ± 6.27	*p* = 0.050	*p* = 0.906	*p* = 0.148
Radiologic stage in JIC						
stage 3A	20 (91%)	17 (100%)	16 (89%)	*p* = 0.495	*p* > 0.999	*p* = 0.486
stage 3B	2 (9%)	0 (0%)	2 (11%)	*p* = 0.495	*p* > 0.999	*p* = 0.486
Locations of osteonecrosis in JIC						
type C1	18 (82%)	15 (88%)	16 (89%)	*p* = 0.679	*p* = 0.673	*p* > 0.999
type C2	4 (18%)	2(12%)	2 (11%)	*p* = 0.679	*p* = 0.673	*p* > 0.999
Collapse of femoral head						
Pre-operation (mm)	1.80 ± 0.23	1.57 ± 0.15	1.51 ± 0.20	*p* = 0.704	*p* = 0.560	*p* = 0.977
Post-operation (mm)	1.24 ± 0.19	1.17 ± 0.16	1.01 ± 0.11	*p* = 0.999	*p* = 0.713	*p* = 0.770
Last follow up (mm)	1.57 ± 0.20	2.49 ± 0.24	2.29 ± 0.28	***p* = 0.023**	*p* = 0.083	*p* = 0.8456
Conversion to THA	3 (14%)	1 (5.9%)	5 (28%)	*p* = 0.618	*p* = 0.430	*p* = 0.177
HHS						
pre-operation	58.5 ± 3.16	57.6 ± 5.26	62.8 ± 4.14	*p* = 0.987	*p* = 0.754	*p* = 0.670
final follow up	95.4 ± 1.26	91.1 ± 3.61	85.4 ± 4.58	*p* = 0.637	*p* = 0.087	*p* = 0.486
P1NP (mg/mL)	69.5 ± 9.52	54.6 ± 6.36	47.4 ± 4.86	*p* = 0.367	*p* = 0.130	*p* = 0.818
TRACP-5b (mU/dL)	378 ± 47.4	385 ± 47.2	343 ± 44.6	*p* = 0.993	*p* = 0.865	*p* = 0.828
BMD(L)(g/cm^2^)	0.99 ± 0.03	0.93 ± 0.03	0.98 ± 0.04	*p* = 0.157	*p* = 0.749	*p* = 0.604
BMD(F)(g/cm^2^)	0.72 ± 0.03	0.77 ± 0.05	0.74 ± 0.02	*p* = 0.614	*p* = 0.948	*p* = 0.839

Data are presented as means± standard error or number (percentage).

GC + TPD, glucocorticoid-associated osteonecrosis of the femoral head (ONFH) treated with perioperative teriparatide; GC, glucocorticoid-associated ONFH treated with perioperative teriparatide; Alc, alcohol-associated ONFH; BMI, body mass index; JIC, Japanese Investigation Committee; THA, total hip arthroplasty; HHS, Harris Hip Score; P1NP, type 1 procollagen-N-propeptide; TRACP-5b, tartrate-resistant acid phosphatase 5b; BMD(L), bone marrow density in the lumbar spine; BMD(F), bone marrow density in the femoral neck.

**Table 2.** Surgical information in each group.

	GCs + TPD (N = 21)	GCs (N = 17)	Alc (N = 18)	GCs + TPD vs GSc	GCs + TPD vs Alc	GCs vs Alc
Operative time (minutes)	106.2 ± 4.09	98.7 ± 4.80	109.1 ± 5.75	*p* = 0.500	*p* = 0.906	*p* = 0.320
Intraoperative bleeding (ml)	96.9 ± 16.0	117 ± 29.9	152 ± 34.5	*p* = 0.851	*p* = 0.308	*p* = 0.641
Femoral neck-shaft angle (pre-operation) (deg)	134 ± 0.93	132 ± 1.13	134 ± 0.87	*p* = 0.325	*p* = 0.992	*p* = 0.321
Femoral neck-shaft angle(post-operation) (deg)	111 ± 1.38	112 ± 1.64	115 ± 1.50	*p* = 0.630	*p* = 0.466	*p* = 0.969
Varus angulation (deg)	22.5 ± 0.86	19.6 ± 0.93	20.1 ± 0.99	*p* = 0.878	*p* = 0.437	*p* = 0.761
Anteversion of femoral neck(pre-operation) (deg)	17.2 ± 2.76	18.3 ± 2.14	18.5 ± 1.89	*p* = 0.944	*P* = 0.919	*p* = 0.998
Anteversion of femoral neck(post-operation) (deg)	20.0 ± 2.61	21.4 ± 2.82	17.6 ± 2.12	*p* = 0.918	*p* = 0.787	*p* = 0.579
ΔAnteversion of femoral neck (deg)	5.06 ± 0.82	5.25 ± 1.03	4.94 ± 0.89	*p* = 0.988	*p* = 0.995	*p* = 0.971
Osteotomy angle against femoral neck (deg)	61.9 ± 4.43	53.5 ± 5.44	63.0 ± 2.39	*p* = 0.359	*p* = 0.980	*p* = 0.297
Osteotomy line on lesser trochanter (%)	55.6 ± 2.21	49.5 ± 3.10	58.8 ± 2.81	*p* = 0.241	*p* = 0.677	*p* = 0.054

Data are presented as means± standard error.

GC + TPD, glucocorticoid-associated osteonecrosis of the femoral head (ONFH) treated with perioperative teriparatide; GC, glucocorticoid-associated ONFH treated with perioperative teriparatide; Alc, alcohol-associated ONFH.

### Effects of teriparatide on bone union and collapse of the femoral head

**Figure 3.**
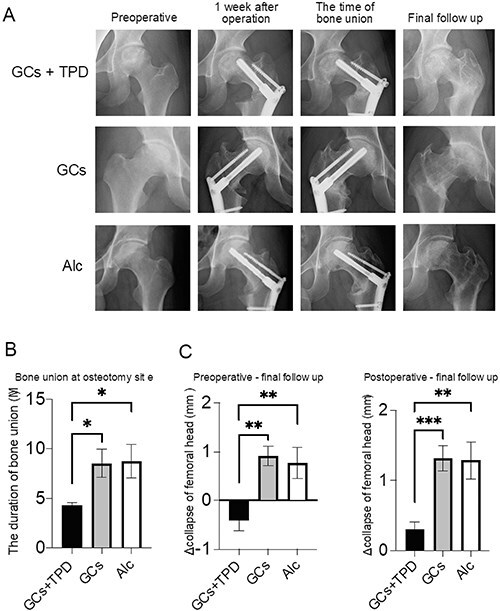
Bone union period evaluation at the osteotomy site. (a) Sequential anteroposterior hip radiographs showing patients with glucocorticoids-associated ONFH treated with (26-year-old woman, GC + TPD) and without teriparatide (27-year-old man, GC and 36-year-old man, Alc), tracking progress from pre-operation to final follow-up. (b) Comparison of bone union period at the osteotomy site among groups, with significant differences marked as *p < 0.05 and **p < 0.01. (c) Changes in femoral head collapse among groups from pre-operation to final follow-up, with significant differences marked as **p < 0.01 and ***p < 0.001.

**Figure 4.**
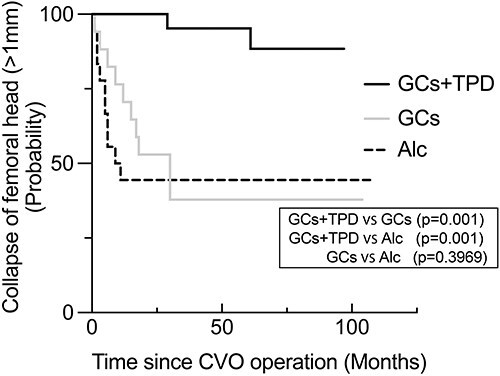
The Kaplan–Meier curves of each group with defined the advanced collapse of the femoral head >1 mm as the advanced collapse, which is the endpoint.

The GC + TPD group exhibited a significantly shorter period of bone union at the osteotomy site than the other two groups (GC + TPD vs GC: 4.3 months vs 8.5 months, *p* = 0.045, GC + TPD vs Alc: 4.3 months vs 8.8 months, *p* = 0.030) (Fig. 3b). In the GC + TPD group, there was a tendency for a larger collapse of the preoperative femoral head, and it significantly decreased compared to the GC group at the final follow-up (*p* = 0.023). (Table 1). However, the progression of femoral head collapse from the preoperative to the final observation and from the postoperative to the final observation was significantly smaller in the GC + TPD group than in the other groups (*p* < 0.001) (Fig. 3c). Kaplan–Meier curves were generated to assess the occurrence of advanced femoral head collapse exceeding 1 mm (Fig. 4). The GC + TPD group (2/21: 9.5%) exhibited a significant reduction in advanced femoral head collapse compared with the GC (10/17: 59%) and Alc groups (10/18: 56%).

### Identifying the contributing factors for the timing of bone union at the osteotomy site

Linear regression analysis was performed to identify the factors associated with the duration of bone union at the osteotomy site (Table 3). Univariate analysis revealed that lower BMI and the use of TPD were significantly associated with a shorter duration of bone union (BMI; *p* = 0.022 and TPD therapy; *p* = 0.004). In multivariate linear regression analysis adjusted for age, sex, BMI, location of osteonecrosis, and use of GCs, the use of TPD was significantly associated with bone union (*p* = 0.005). However, parameters related to surgical correction did not emerge as contributing factors to bone union in both univariate and multivariate analyses.

**Table 3.** Univariate and multivariate linear regression analyses of the timing of bone union at the osteotomy site.

	Univariate				Multivariate			
Variables	standardized β	SE	*t* value	*p* value	standardized β	SE	*t* value	*p* value
Age	0.067	0.076	0.501	0.618				
Sex	0.026	1.561	0.197	0.845				
BMI	0.322	0.218	2.237	**0.022**				
Locations of osteonecrosis in JIC	0.061	1.025	0.476	0.636				
Use of glucocorticoids	−0.215	1.580	1.622	0.111				
P1NP	−0.161	0.003	0.917	0.365	−0.260	0.046	1.054	0.2.99
TRACP5b	−0.107	0.006	0.605	0.549	−0.004	0.007	0.021	0.993
Varus angulation	−0.100	0.183	0.744	0.460	−0.125	0.216	0.778	0.441
ΔAnteversion of femoral neck	0.052	0.255	0.328	0.744	0.055	0.255	0.345	0.732
Osteotomy angle against femoral neck	−0.071	0.050	0.482	0.632	−0.158	0.056	0.958	0.344
Osteotomy line on lesser trochanter	0.091	0.064	0.675	0.503	0.050	0.071	0.330	0.745
TPD therapy	−0.374	1.444	3.014	**0.004**	−0.474	1.851	2.978	**0.005**

Multivariate analyses were adjusted for sex, age, BMI, location of osteonecrosis on the JIC, and the use of glucocorticoids. SE: standard error.

BMI, body mass index; JIC, Japanese Investigation Committee; P1NP, type 1 procollagen-N-propeptide; TRACP-5b, artrate-resistant acid phosphatase 5b; Danteversion of the femoral neck, Change in anteversion of the femoral neck between preparation and postoperatively; TPD, teriparatide.

### Identifying contributing factors on the advanced femoral head collapse

Univariate analysis identified the duration of bone union at the osteotomy site and the use of TPD as contributing factors to the advanced progressive collapse of the femoral head exceeding 1 mm (Table 4). These two parameters were also identified as potential contributing factors to advanced femoral head collapse in a Cox proportional hazards model adjusted for age, sex, BMI, and location of osteonecrosis, which could affect the collapse in previous reports. No parameters related to surgical correction were identified as contributing factors to advanced femoral head collapse in both univariate and multivariate Cox regression analyses.

**Table 4.** Cox regression analysis for advanced collapse of the femoral head > 1 mm.

	Univariate analysis			Multivariate analysis		
Variables	HR	SE	*p* value	HR	SE	*p* value
Age	1.011	0.021	0.615			
Sex	1.167	0.444	0.727			
BMI	1.019	0.066	0.777			
Locations of osteonecrosis in JIC	1.788	0.509	0.254			
BMD(L)	0.258	1.946	0.487	0.458	2.076	0.707
BMD(F)	2.354	1.650	0.604	2.483	2.054	0.658
Varus angulation	1.041	0.052	0.442	1.009	0.063	0.893
ΔAnteversion of femoral neck	1.063	0.063	0.327	1.067	0.070	0.356
Osteotomy angle against femoral neck	1.008	0.014	0.588	1.002	0.017	0.914
Osteotomy line on lesser trochanter	0.999	0.019	0.959	0.977	0.022	0.286
Period of bone union	1.058	0.026	**0.029**	1.089	0.034	**0.011**
TPD therapy	0.103	0.747	**0.002**	0.075	0.778	**0.001**

Multivariate analyses were adjusted for sex, age, BMI, location of osteonecrosis on the JIC.

HR, hazard ratio; SE, standard error; BMI, body mass index; JIC, Japanese Investigation Committee; BMD(L), bone marrow density in the lumbar spine; BMD(F), bone marrow density in the femoral neck; Danteversion of the femoral neck, Change in anteversion of the femoral neck between preparation and postoperatively; TPD, teriparatide.

### The variability in inter- and intra-observer measurements

The inter- and intra-observer variability in the collapse of the femoral head was assessed using Cohen’s kappa coefficient, yielding values of 0.745 (moderate) and 0.830 (good), respectively.

## Discussion

The results of this retrospective study demonstrate that TPD has a positive impact on bone union at the osteotomy site and prevents the progression of femoral head collapse following joint-preserving osteotomy. Previous clinical knowledge of the efficacy of TPD in post-osteotomy bone healing is limited, with only a few case reports available [31, 32]. This study did not find any significant differences in the duration of bone union between the GCs and Alc groups. However, the average duration of bone union was longer in these groups than in trochanteric fractures treated with a sliding hip screw, as reported in a previous study [33] (8 months vs. 3 months), indicating a slower bone union process in joint-preserving osteotomy for ONFH. Additionally, information on the effectiveness of bone metabolism regulators in preventing femoral head collapse in patients undergoing joint-preserving osteotomy is limited. Although the short- and mid-term clinical outcomes did not show significant differences, the use of TPD in joint-preserving osteotomy for ONFH may be beneficial in terms of promoting bone union and preventing the progression of collapse.

This study found that TPD administration halved the bone healing duration, consistent with prior reports from animal osteotomy models [34] and human clinical fracture studies [24]. The multivariate linear regression analysis findings, highlighting TPD administration as an independent bone healing factor, further supported its usefulness for osteotomy. The degree of femur correction and the anatomical features in this study did not affect the bone-healing period. Although earlier studies have reported low bone fusion failure rates in CVO [6, 35], the factors linked to bone fusion and its duration remain unclear. Therefore, more comprehensive observational studies are needed to identify the factors associated with osteotomy bone healing.

The effectiveness of preventing femoral head collapse in this study aligns with that of previous reports, supporting the usefulness of TPD in conservative ONFH treatment [25]. Teriparatide is an osteoporosis drug that stimulates osteoblasts and promotes bone formation and fracture healing, and it has a stronger effect on increasing bone mass as a treatment against bone fragility than other osteoporosis drugs [36, 37]. Studies have demonstrated that bone remodeling abnormalities at the boundary between necrotic and healthy areas contribute to femoral head collapse [13, 38]. In a steroid-associated osteonecrosis rat model, TPD administration suppressed osteonecrosis and enhanced femoral head bone strength [39]. Consequently, TPD might be effective not only in osteotomy site bone union but also in normalizing bone remodeling at the boundary between necrotic and healthy areas, thus preventing femoral head collapse. The finding that the bone union period at the osteotomy site was also an independent factor for femoral head collapse supports this possibility.

This study has several limitations. The method used to measure femoral head collapse may not be accurate, as it was determined by measuring the distance along a line passing through the femoral head center, potentially failing to accurately capture the weight-bearing area collapse length. However, standardization of the method is essential to ensure reliable measurements, and the usefulness of this method for evaluating bone head crushing has been reported in the past [40]. This measurement technique has shown effective results with minimal inter- and intra-observer variability. The sample size in this current study was small because ONFH cases eligible for CVO surgery are extremely rare. Further long-term follow-ups and larger multicenter studies are required for a comprehensive evaluation of collapse and THA conversion. However, this study offers valuable insights into early bone-healing effects and femoral head collapse in real-world human osteotomies using TPD.

In conclusion, the administration of TPD enhanced bone formation at the surgical osteotomy site and reduced femoral head collapse within the early postoperative period. Therefore, combining curved varus osteotomy and TPD may be an effective management strategy for femoral head osteonecrosis in younger patients with glucocorticoid-associated osteoporosis.

## Data Availability

The datasets generated during and/or analyzed during the current study are available from the corresponding author on reasonable request.
